# Effects of Natural Hirudin and Low Molecular Weight Heparin in Preventing Deep Venous Thrombosis in Aged Patients with Intertrochanteric Fracture

**DOI:** 10.1038/s41598-018-27243-1

**Published:** 2018-06-11

**Authors:** Zhengdong Zhang, Zheng Li, Jun Li, Lei Liu

**Affiliations:** 1 Department of Othopedics, West China Hospital, Sichuan University, 37# Wainan Guoxue Road, Chendu, 610041 People’s Republic of China; 2 Department of Othopedics, The second People’s Hospital of Jiu Long Po District, Chongqing, 400050 People’s Republic of China; 3 The People’s Hospital of Jiu Long Po District, Chongqing, 400050 People’s Republic of China

## Abstract

Our objective was to evaluate the efficacy and safety of natural hirudin and low molecular weight heparin (LMWH) in the prevention of perioperative deep venous thrombosis (DVT) in elderly patients with intertrochanteric fracture. From June 2014 to June 2017, 96 patients with intertrochanteric fractures were treated with proximal femoral nail antirotation (PFNA) were randomly divided into two groups. For DVT prevention, 45 patients were treated with oral natural hirudin and subcutaneous LMWH-calcium (test group) and 51 patients were treated with subcutaneous LMWH-calcium (control group). The mean intraoperative bleeding, wound drainage and incisional hematoma were higher in the test group, with no significant differences between the groups. There were significant differences in distal intramuscular venous thrombosis (*P* = 0.043). Both activated partial thromboplastin time (APTT), thrombin time (TT), and prothrombin time (PT) lengthened in both groups postoperatively, and there was a significant difference between the two groups two weeks postoperatively. D-dimer were significantly different and platelet count (PLT) did not differ between groups two weeks postoperatively. In elderly patients with unilateral intertrochanteric fracture after PFNA on anticoagulant therapy, the combination of natural hirudin and LMWH was more effective than that of LMWH-calcium alone, with no significant difference with regard to safety.

## Introduction

There is a high incidence of hip fracture in elderly patients, and deep venous thromboembolism (DVT) is one of the most common complications after hip fracture surgery^1^. It has been reported that the incidence of DVT after hip fracture is 46–60%^2,3^, and its occurrence could lead to post thrombotic syndrome (PTS) and a fatal pulmonary embolism (PE). At present, factor Xa inhibitors (e.g., heparin), vitamin K antagonists, direct thrombin inhibitors, and mechanical pressure are all effective in the prevention and treatment of DVT in patients with hip fractures. However, there are some limitations to their use, including hemorrhage^4^, heparin-induced thrombocytopenia (HIT)^5^, increased liver and kidney function loads^6^, poor prevention of thrombosis^7^, and poor patient compliance^8,9^. Therefore, it is necessary to seek medications for DVT prevention after hip fracture that have less side effects and are associated with better patient compliance. Natural hirudin, a hirudin isolated from the saliva of bloodsucking leeches, has been used in China for many years and has significant anticoagulant effects^10–12^. We used oral natural hirudin combined with subcutaneous low molecular weight heparin calcium (LMWH-calcium) for the perioperative treatment of senile intertrochanteric fractures to prevent DVT, and observed its safety and effectiveness.

## Results

### Baseline Characteristics

The baseline characteristics of the patients in both groups are summarized in Table 1. There was no significant difference in sex, age, fracture site (left/right), or Caprini scores between the test and control groups (*P* > 0.05).

**Table 1:** Comparison of the baseline data of the patients in the control and test groups.

	Control group n = 51	Test group n = 45	*P* value
Age (years)	69.98 ± 5.35	69.58 ± 5.22	0.711^a^
Sex (men/women)	23/28	19/26	0.777^b^
BMI (kg/m^2^)	23.34 ± 1.96	23.35 ± 2.06	0.967^a^
Affected side (left/right)	22/29	23/22	0.435^b^
Caprini score	10.41 ± 1.13	10.37 ± 1.13	0.689^a^

The control group received daily subcutaneous injections of LMWH 2000 IU, and the test group received lyophilized natural hirudin powder in oral enteric-coated capsule form, combined with LMWH subcutaneous injection. ^a^Independent *t* test. ^b^Chi-square test. The *P* values shown are for inter-group comparisons. BMI: Body mass index, LMWH: Low molecular weight heparin.

### Efficacy

There was no significant difference between the two groups in the intraoperative blood loss or the postoperative volume of incision drainage, but the total blood loss of the test group was increased compared to the control group (*P* > 0.05) (Table 2).

**Table 2:** Comparison of clinical outcomes between the control and test groups.

Variables	Control group n = 51 (*P* of normality test)	Test group n = 45 (*P* of normality test)	*P* value
Intraoperative bleeding (ml)	345.7 ± 48.26	362.2 ± 73.20	0.202^*^
Postoperative drainage (ml)	42.75 ± 12.66	44.44 ± 13.57	0.527^*^
Incisional hematoma	2	4	0.414^*^
Distal intramuscular VTE	9	2	0.043^*^
Proximal DVT	3	1	0.701^*^
Activated Partial Thromboplastin time (APTT in seconds)			
On admission	27.60 ± 2.41(0.081)	27.56 ± 2.47(0.200)	0.947^#^
Two days after the operation	27.84 ± 2.64(0.027)	28.29 ± 2.78(0.049)	0.419^#^
One week after the operation	28.45 ± 2.62(0.018)	30.13 ± 2.64(0.200)	0.004^#^
Two weeks after the operation	28.49 ± 2.83(0.200)	30.49 ± 2.07(0.024)	0.001^#^
Thrombin time (TT in seconds)			
On admission	12.27 ± 2.23(0.018)	12.28 ± 1.42(0.006)	0.958^#^
Two days after the operation	12.17 ± 1.79(0.001)	12.40 ± 1.23(0.017)	0.691^#^
One week after the operation	12.82 ± 1.84(<0.001)	14.40 ± 1.49(<0.001)	<0.0001^#^
Two weeks after the operation	13.31 ± 1.54(<0.001)	14.80 ± 1.14(<0.001)	<0.0001^#^
Prothrombin Time (PT in seconds)			
On admission	11.06 ± 0.88(0.200)	11.10 ± 1.05(0.164)	0.866^#^
Two days after the operation	11.28 ± 0.88(0.200)	11.32 ± 0.92(0.200)	0.127^#^
One week after the operation	11.44 ± 0.93(0.200)	11.75 ± 0.92(0.200)	0.113^#^
Two weeks after the operation	11.54 ± 1.00(0.038)	12.03 ± 0.93(0.200)	0.017^#^
D-dimer (µg/ml)			
On admission	0.598 ± 0.241(0.008)	0.589 ± 0.255(0.067)	0.693^#^
Two days after the operation	0.610 ± 0.180(0.200)	0.566 ± 0.207(0.192)	0.266^#^
One week after the operation	0.408 ± 0.137(0.200)	0.234 ± 0.102(0.200)	<0.0001^#^
Two weeks after the operation	0.315 ± 0.138(0.054)	0.184 ± 0.796(0.006)	<0.0001^#^
Blood platelets (PLT × 10^9^/L)			
On admission	238.41 ± 42.08(0.200)	236.98 ± 46.89(0.200)	0.875^#^
Two days after the operation	227.24 ± 36.33(0.191)	223.36 ± 48.21(0.200)	0.655^#^
One week after the operation	215.63 ± 38.38(0.200)	216.53 ± 40.77(0.191)	0.911^#^
Two weeks after the operation	207.51 ± 37.75(0.200)	200.49 ± 38.56(0.200)	0.370^#^

^*^Independent *t* test or chi-square test. ^#^Comparison of activated partial thromboplastin time (APTT), thrombin time (TT), prothrombin time (PT), D-dimer and platelet (PLT) count between the control and the test group, evaluated using the paired multivariate analysis of variance or Wilcoxon rank test. The *P* values shown are for inter-group comparisons. VTE: Venous thromboembolism. DVT: Deep venous thrombosis.

In terms of postoperative hematomas, although the incidence of incisional hematomas in the test group was 8.8% (4/45) and in the control group was 3.9% (2/51), the difference between the groups was not statistically significant. There were no episodes of intracranial hemorrhage, retroperitoneal hemorrhage, melena or hematochezia, hematuria, or need of blood transfusions. The diameter of the hematomas in all incisions was less than 5 cm, and all were cured by drainage or aspiration (Table 2).

Before anticoagulant therapy, we measured the activated partial thromboplastin time (APTT), thrombin time (TT), prothrombin time (PT), D-dimer, platelet (PLT) count at baseline, none which were statistically significant when compared between the two groups (*P* > 0.05). Two weeks postoperatively, the APTT, TT and PT in both groups were longer than the pre-treatment times. The prolongation in the test group was greater than that in the control group, and differences were statistically significant (*P* < 0.05). Additionally, the D-dimer levels in both groups were significantly lower than the pre-treatment values. The decrease in the test group was significantly greater than that in the control group, and the difference was significant (*P* < 0.05). The PLT counts of the two groups were lower than that the pre-treatment counts. There was no significant difference between the two groups (*P* > 0.05), and both groups counts were within the normal range.

In terms of venous thromboembolism (VTE), all DVTs were detected on ultrasound two weeks postoperatively. There was no significant difference in proximal DVT between the two groups (test group 2.2% (1/45) and 5.8% (3/51), respectively, with *P* > 0.05), But in the case of distal intramuscular VTEs, there was a significant difference between the two groups of patients (test group 4.4% (2/45) and 17.6% (9/51), respectively, with *P* < 0.05). All patients diagnosed with thrombus were treated with thrombolytic therapy, and the follow-up ultrasounds showed that the thrombi had disappeared. (Table 2).

The patients were followed up by outpatient clinic visits or via telephone for at least 3 months after discharge, but 9 of them were lost to follow-up for other reasons. Another patient, at 2 weeks after discharge accidentally fell again which lead to a broken intramedullary nail requiring re-admission and surgery. No symptomatic thrombi occurred in any of the patients who were abtained to followe up after discharge.

## Discussion

Elderly patients with femoral trochanteric fracture due to impaired functioning of important organs may have local bleeding or swelling caused by trauma. In these patients, pain limits the activities of the affected limbs and the blood flow is relatively weak in the lower limbs. Additionally, these patients are in a hypercoagulable state after fracture. The combination of these factors leads to the Virchow’s triad effect, which increases the risk of VTEs^13^. If the thrombus detaches and embolizes, there is a high risk of fatal consequences such as PE, which is the main cause of death in elderly patients after hip fractures^1^. More than 40% of patients with hip fractures have subsequent DVTs, and at least 11% develop PEs^14^. It has been suggested that the incidence of DVT after hip fracture in the Asian population is lower than that in Western populations^15^. But later studies showed that the incidence of DVT after hip fracture in elderly patients in Asia is similar to that in Western patient populations^2,16,17^. Therefore, it is important to prevent the occurrence of DVT in elderly patients with hip fracture.

LMWH is the preferred anticoagulant recommended in many guidelines^18–20^, and its use is well-known and accepted by the vast majority of doctors. Other anticoagulant options include warfarin, rivaroxaban, and dabigatran^21,22^. However, the actual clinical results are not as expected, and the best anticoagulant drugs and treatments are still unknown.

Natural hirudin is a natural, selective, direct thrombin inhibitor^23^. It is composed of 65 amino acid residues, and its molecular weight is about 7 kDa. Unlike natural hirudin, recombinant hirudin has no sulfate group on the tyrosine at the 63^rd^ position^24^. Both forms have proved to be effective anticoagulants in various experimental models of thrombus formation and their clinical applications^25–28^. The hirudins have achieved good results in the treatment of HIT and are recommended by the United States Food and Drug Administration (FDA) as anticoagulants for use in percutaneous coronary intervention (PCI)^29^. In China, natural hirudin freeze-dried powder, a traditional Chinese medicine, has been used as an auxiliary anticoagulant for many years^10–12^. Hirudin is rapidly inactivated by pepsin after oral administration, but trypsin and chymotrypsin do not destroy its activity. Therefore, we used enteric-coated capsules to avoid this gastrointestinal reaction effect^11^.

At present, there are no large-scale, comparative studies of hip fractures in elderly patients using natural hirudin and LMWH in combination. Therefore, we can only compare the results of this study with those that evaluated patients with hip fractures treated with LMWH. Many scholars have confirmed the objective validity of the Caprini score in clinical applications^30^. The Caprini scoring system classifies patients with scores ≥5 as patients at high-risk for VTE. To verify the effectiveness of this study, we included elderly patients with intertrochanteric fractures and Caprini scores of 9–12. Considering the high incidence of preoperative DVT in patients not receiving anticoagulants, we began routine thromboprophylaxis between the time of admission to 12 hours before surgery^20^. The incidence of proximal thrombus formation in the test group and the control group was 2.2% (1/45) vs. 5.8% (3/51), respectively (*P* = 0.701). However, there was a significant difference in the incidence of distal intermuscular venous thrombosis 4.4% (2/45) vs. 17.6% (9/51), respectively (*P* = 0.043). In our study, the incidence of distal intermuscular venous thrombosis in the LMWH group was higher than that reported (5.7%) by Long *et al*.^31^, but they included any patients over 18 years old with hip fractures, as opposed to just elderly with hip fractures in this study. We only included elderly patients with Caprini scores greater than or equal to 9, and less than or equal to 12. Among the patients we included, all distal and proximal venous thrombi were asymptomatic. At the same time, we observed that in the test group there was a reduction in the incidence of distal intermuscular venous thrombosis, but there was no significant difference in the incidence of proximal venous thrombosis between the two groups. Lee *et al*. proved that distal intermuscular VTE is usually stable^32^, which do not cause severe pulmonary thromboembolism normally. Distal intermuscular VTE, at the same time, is hard to migrate into proximal vein when patients undergoing anticoagulation therapy. Consequently, it is not clear how much benefit a patient really gets from decreasing the incidence of distal intermuscular VTE^33,34^.

The D-dimer level is also a risk factor for VTE^35,36^. We obtained continuous D-dimer measurements. Upon hospital admission, both groups of patients had higher baseline D-dimer levels, but no evidence of thrombus on color Doppler ultrasound. We could not exclude the normal increase in D-dimer levels after fracture^37^, and there was no significant difference between the two groups (*P* = 0.693), which is similar to the results of Luksameearunothai’s research^38^. At 1 and 2 weeks after the surgery, the values of D-dimer were significantly decreased in both groups, and the comparison between the groups was significant (*P* < 0.001). Our results showed that the D-dimer level in the test group decreased faster than that of the control group. Considering the sensitivity and specificity of D-dimers, we only monitored the value of D-dimer as a reference index, that is, we performed vascular ultrasound examinations for patients with sustained high D-dimer levels.

In our results, we found that the APTT, TT and PT values of the patients in the test group were significantly different from those in the control group at two weeks after the operation (*P* = 0.001, *P* < 0.0001 and *P* = 0.017, respectively). This result is similar to that of Dong *et al*.^11^. Hirudin as a direct thrombin inhibitor, prolongs both the APTT, TT and PT. There were no patients that had prolongation of APTT, TT or PT beyond the normal ranges. Heparins have been found to reduce platelet counts and induce HIT^5^. In our study, no patient had thrombocytopenia beyond the lower limit of the normal range and there were no statistically significant differences in thrombocytopenia between the two groups at two weeks after surgery (*P* = 0.370). Mumoli *et al.*^39^ reported successful experiences with the use of recombinant hirudin for the treatment of HIT with pulmonary embolism. Joseph L *et al*.^29^ considered that recombinant hirudin (Bivalirudin) was an effective and safe alternative anticoagulant to treat HIT with or without thrombus. We speculated that natural hirudin could prevent the occurrence of heparin-like thrombocytopenia when LMWH-calcium was used, however, there were no incidents of suspected HIT in either group of patients in our study.

As a complication of chemical prevention, bleeding events are also a matter of concern to surgeons^40^. The test and control groups in our study had intraoperative blood losses of 362.2 ± 73.2 ml and 345.7 ± 48.3 ml, respectively (*P* = 0.202). The mean postoperative wound drainages were 44.4 ± 13.6 ml in the test group and 42.75 ± 12 ml in the control group, and there were no significant differences between groups (*P* = 0.527). Postoperative hematoma occurrences were 8.8% (4/45) in the test group, and 3.9% (2/51) in the control group (*P* = 0.414). We observed that the amount of intraoperative blood loss, incisional drainage, and hematoma incidence in the test group were greater than those in the control group, but there was no statistically significant difference between the two groups. There was no associated massive hemorrhage in either groups. The incidence of slight hemorrhage (diameter of hematoma <5 cm) in the control group was less than Fuji *et al*.^6^ reported 10.3% and the results are similar to those of Liu *et al*.^41^. We believe that this difference is related to the minimally invasive nature of the procedure.

Our research has several limitations. First, this was a single-center retrospective analysis. The sample size was relatively small; therefore, prospective randomized controlled trials will be needed in the future to include more patients. Second, considering the risk of venography, we used Doppler ultrasound to examine the blood vessels, but the true accuracy of Doppler ultrasound for diagnosing thrombus may not be as good as that of venography. Third, we re-examined the patients after discharge, but we did not routinely perform routine venography or ultrasound examination. We only performed ultrasound examinations of the lower limb veins in patients suspected to have VTE on the basis of clinical symptoms. But none of the patients underwent ultrasound examinations during follow ups because none of them developed clinical signs or symptoms of VTE. Therefore, the true incidence of VTE may be underestimated. After discharge from the hospital, the patients had to receive their doses of LMWH in community or outpatient clinics and administer the medicines to themselves at home. This may have led to irregular or unreliable medication protocols. Finally, although there was no significant statistical difference between the demographic and clinical characteristics of the population in this study, it is possible that there were unmeasurable confounding factors leading to a deviation of the results. Despite these shortcomings, to our knowledge, this is the first study to report a combination of natural hirudin enteric capsule oral and LMWH subcutaneous injection for the prevention of DVT during the perioperative period of PFNA surgery for hip fractures in elderly individuals.

In summary, in elderly patients who suffered intertrochanteric fractures with Caprini scores of 9–12 undergoing PFNA surgery, the combination of lyophilized natural hirudin powder oral enteric-coated capsule combined with subcutaneous injections of LMWH was more effective than LMWH-calcium alone, but there was no significant difference in safety.

## Materials and Methods

### Study Patients

This was a retrospective cohort study conducted by the Department of Orthopedics, the second People’s Hospital of Jiu Long Po District of Chongqing, People’s Republic of China and approved by the Internal Research Management and Ethics Committees. Written informed consent from all the participants was obtained. The clinical study was conducted in accordance with the Declaration of Helsinki principles.

During the data collection process, 127 cases of senile intertrochanteric fracture that underwent proximal femoral nail antirotation (PFNA) surgery met the inclusion criteria which were: (1) age >60 years of either sex; (2) weight range 45–100 kg; (3) suffered low energy injuries caused by fall, with neither additional site fractures nor open fractures; (4) no coagulation dysfunction or history of thromboembolism within 6 months prior to admission; (5) no history of internal implant surgery, history of diabetes, oral anticoagulants, or long-term use of hormone therapy, and no history of drug/alcohol/cigarette abuse; (6) bilateral lower extremity vascular ultrasound, cervical vascular ultrasound and abdominal large vessel ultrasound with negative results upon hospital admission; (7) non-pathological fractures treated with PFNA after admission and operated on within 1–3 days after injury; (8) general anesthesia used in all operations and no history of tranexamic acid used during any previous surgery; (9) liver and kidney function, blood routine, coagulation function were not abnormal on admission, (10) no history of allergies to heparins and hirudins; (11) in addition to the above conditions, the Caprini scores needed to be ≥5 and ≤ 12, respectively.

Thirty-one patients were excluded because they did not meet the criteria. Finally, 96 patients were included in the study cohort, 51 received LMWH subcutaneous injections (control group), and 45 received lyophilized natural hirudin powder in enteric-coated capsule form combined with LMWH subcutaneous injections (test group).

### Drugs

Lyophilized natural hirudin powder in enteric-coated capsule form was manufactured by Chongqing Duoputai Pharmaceutical Co., Ltd. Chongqing, China (Brand name: Maixuekang, 0.25 g/capsule, Patent number ZL201010595610.2, Medicine Permission Number Z10970056). Enoxaparin as LMWH-calcium was manufactured by Sanofi-Aventis Co., Ltd. Beijing, China, (Brand name: Kesai, 0.2 ml, 2000 Axa IU/branch).

### Study Design

The test group was administered the lyophilized natural hirudin enteric-coated capsule (three capsules orally, three times daily, for a total of 2.25 grams per day) in combination with a prophylactic dose of 0.2 ml LMWH-calcium administered via subcutaneous injection once daily. The control group received 2000 IU of LMWH-calcium injected subcutaneously once per day. After admission, the test and control group patients completed examinations to prove that there were no anticoagulant side effects or contraindications, and the hirudin/LMWH-calcium and LMWH-calcium therapies, respectively, were administered until 12 hours before surgery.

All patients were given inhaled general anesthesia, were placed in the supine position on a traction surgical bed, and closed reductions via minimally invasive internal fixations with PFNA were performed. The operative times were usually 30–60 minutes. Single doses of first- or second-generation antibiotics were administered intraoperatively and postoperatively. A non-negative pressure drainage tube was routinely placed in the proximal femoral trochanter incision after surgery and the drainage tubes were generally removed 1–2 days postoperatively. At 12 hours after surgery, the hirudin/LMWH-calcium regimen for the test group and the LMWH-calcium alone regimen for the control group were restarted and continued for a total of 4 weeks. All patients were instructed to perform active and passive functional exercises as soon as possible after surgery, including quadriceps contraction exercises and hip, knee, and ankle exercises without loads. The use of foot pressure pumps began on the first night after surgery. If venous thromboembolism (VTE) was confirmed, anticoagulant therapy was discontinued and routine thrombolytic therapy was initiated. Patients were generally discharged two weeks after surgery and continued to receive their respective medication regimens in community or outpatient clinics.

### Evaluation Methods

On admission, on the second postoperative day, 1 week after surgery, and 2 weeks after surgery, routine blood work was examined, including coagulation indices and D-dimer levels. Immediately after admission and each week after surgery, bilateral lower extremity vascular ultrasounds, cervical vascular ultrasounds, and abdominal large vessel ultrasounds were performed until the patients were discharged, and we documented the presence of any asymptomatic thrombi. Chest computed tomography (CT) examinations were performed for any suspicion of PE. We documented all intraoperative incisional bleeding and postoperative incisional drainage. We identified and assessed any suspected major hemorrhage as defined by the International Society for Thrombosis and Hemostasis (ISTH) − that is, fatal hemorrhage, a decrease of ≥2 g/dL in the hemoglobin level, blood transfusion requiring ≥2 U pRBCs, bleeding from major organs, or the occurrence of major clinically associated non-massive hemorrhage events.

### Statistical Analysis

The statistical analysis was performed using Statistical Package of Social Sciences (SPSS, IBM Corp.) software version 19.0. The basic characteristics of the patients were compared using the chi-square test of classified variables. Disaggregated data were expressed as frequencies or percentages, and continuous data were presented as means and standard deviations. We used the Kolmogorov-Smirnov to test normality, and the paired *t* test and Wilcoxon rank test were used to compare intra-group continuous variables with or without normal distributions, respectively. The paired multivariate analysis of variance or the Wilcoxon rank test was used to compare inter-group continuous variables with or without normal distributions, respectively. *P* values < 0.05 were considered statistically significant.

### Data availability statement

The datasets generated and analyzed during the current study are available from the corresponding author on reasonable request.
